# Gestation and breastfeeding in schistosomotic mothers differently modulate the immune response of adult offspring to postnatal *Schistosoma mansoni* infection

**DOI:** 10.1590/0074-02760150293

**Published:** 2016-02

**Authors:** Patrícia d‘Emery Alves Santos, Virgínia Maria Barros de Lorena, Érica de Souza Fernandes, Iana Rafaela Fernandes Sales, Wheverton Ricardo Correia do Nascimento, Yara de Miranda Gomes, Mônica Camelo Pessoa de Azevedo Albuquerque, Vlaudia Maria Assis Costa, Valdênia Maria Oliveira de Souza

**Affiliations:** 1 Universidade Federal de Pernambuco, Laboratório de Imunopatologia Keizo Asami, Setor de Imunologia, Recife, PE, Brasil; 2 Fundação Oswaldo Cruz,Centro de Pesquisas Aggeu Magalhães, Recife, PE, Brasil; 3 Universidade Federal de Pernambuco, Centro de Ciências da Saúde, Departamento de Medicina Tropical, Recife, PE, Brasil

**Keywords:** schistosomiasis, pregnancy, breastfeeding, postnatal infection, ovalbumin

## Abstract

*Schistosoma mansoni* antigens in the early life alter homologous and heterologous immunity during postnatal infections. We evaluate the immunity to parasite antigens and ovalbumin (OA) in adult mice born/suckled by schistosomotic mothers. Newborns were divided into: born (BIM), suckled (SIM) or born/suckled (BSIM) in schistosomotic mothers, and animals from noninfected mothers (control). When adults, the mice were infected and compared the hepatic granuloma size and cellularity. Some animals were OA + adjuvant immunised. We evaluated hypersensitivity reactions (HR), antibodies levels (IgG1/IgG2a) anti-soluble egg antigen and anti-soluble worm antigen preparation, and anti-OA, cytokine production, and CD4^+^FoxP3^+^T-cells by splenocytes. Compared to control group, BIM mice showed a greater quantity of granulomas and collagen deposition, whereas SIM and BSIM presented smaller granulomas. BSIM group exhibited the lowest levels of anti-parasite antibodies. For anti-OA immunity, immediate HR was suppressed in all groups, with greater intensity in SIM mice accompanied of the remarkable level of basal CD4^+^FoxP3^+^T-cells. BIM and SIM groups produced less interleukin (IL)-4 and interferon (IFN)-*g*. In BSIM, there was higher production of IL-10 and IFN-*g,* but lower levels of IL-4 and CD4^+^FoxP3^+^T-cells. Thus, pregnancy in schistosomotic mothers intensified hepatic fibrosis, whereas breastfeeding diminished granulomas in descendants. Separately, pregnancy and breastfeeding could suppress heterologous immunity; however, when combined, the responses could be partially restored in infected descendants.

Schistosomiasis is endemic in 78 countries and at least 261 million people are estimated to be infected worldwide (WHO 2015). In Brazil, the only known causative species is *Schistosoma mansoni* (do Amaral et al. 2006). The infection is chronic because of *S. mansoni* eggs, which remain lodged in intestinal and hepatic tissues and provoke typical eosinophilic inflammation that later becomes dominated by fibrotic deposits (Gryseels et al. 2006). However, in the chronic phase, most patients living in endemic areas are asymptomatic (only 4-12% show severe manifestations of the disease) (Caldas et al. 2008) due to an immunomodulatory phenomenon. The initial T-helper (Th)1 immune response [interferon (IFN)-g, interleukin (IL)-2 and IgG2a] against infective larvae antigens is inhibited on the 60th day post-infection (dpi) by a predominant Th2 immune response, with the production of IL-4, IL-5, and IL-10 that are induced by egg antigens and promote the IgE and IgG1 production (Finkelman et al. 1991, Pearce et al. 1991, Moore et al. 2001). This response is associated with the stimulation of T regulatory (Treg) cells and IL-10, which control the granulomatous reaction around eggs in the host (McKee & Pearce 2004). Studies in animals and humans have shown that this immunosuppressive profile extends to heterologous antigens. There are reduced Th1 and Th17 responses to viral, bacterial, and self-antigens (Actor et al. 1993, Sabin et al. 1996, La Flamme et al. 2003, Osada et al. 2009, Ruyssers et al. 2010), and Th2 responses to allergens have also been shown to be attenuated (Medeiros Jr et al. 2003, Smits et al. 2007).

There are approximately 40 million women of fertile age, including 10 million pregnant women who are chronically infected by *Schistosoma* in endemic areas (Friedman et al. 2007, Hillier et al. 2008). The consequences of maternal infection on the immune systems of descendants have been the subject of investigation. For responses to homologous antigens, the immunomodulation phenomenon appears to be maintained in postnatal infections. It is related to a granulomatous response to the eggs of*S. mansoni,* which are less frequent or even absent in mice that are born and suckled by schistosomotic mothers. The expression levels of the IL-12 and transforming growth factor (TGF)-β genes, which encode cytokines with potential inflammatory and regulatory activities, are significantly higher in animals exposed to prenatal infection (Lenzi et al. 1987, Attallah et al. 2006, Othman et al. 2010). However, whether this protection from a granulomatous reaction in adult descendants is established during pregnancy or by breastfeeding from an infected mother remains unclear. Likewise, it is not known whether negative modulation of immune responses to heterologous antigens is preserved in postnatal infections of mice that were born to or breastfed by infected mothers.

To address these questions, after adoptive breastfeeding, mice were divided into the following groups: born (BIM), suckled (SIM), or born/suckled (BSIM) in schistosomotic mothers, as well as animals born/suckled by noninfected mothers (control). When adults, the animals were infected and some of them were immunised with ovalbumin (OA) in adjuvant. We found that offspring from *S. mansoni*-infected mothers that were then breastfed by noninfected mothers presented a granulomatous reaction worsened, which was strongly ameliorated by breast milk from these mothers. Related to the response to OA during postnatal infections, previous exposure to parasitic antigens*in utero* or through breast milk diminishes the heterologous response, favoring strong anti-OA immunosuppressive potential. However, when associated with pregnancy followed by breastfeeding, heterologous immunity in the infected descendants is partially restored.

## MATERIALS AND METHODS

**Fig. 1:**
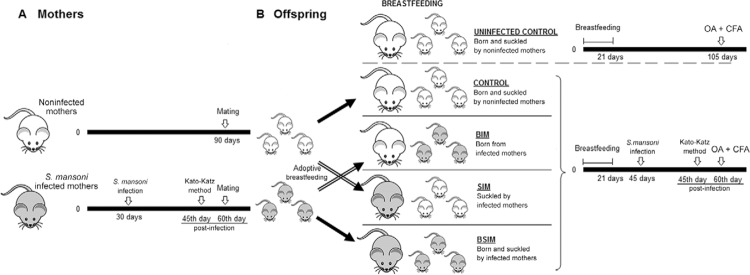
: experimental design. Noninfected or infected (20*Schistosoma mansoni* cercariae) Swiss Webster female mice were caged with male mice at a 1:1 ratio for mating (A). Immediately after birth, offspring mice born from infected mothers (BIM) were suckled by noninfected mothers and offspring of noninfected mothers were suckled by infected mothers (SIM). Another groups born and suckled by infected mothers (BSIM) or noninfected females (control) were also suckled by their own mothers. Six-week-old male offspring were infected (80 *S. mansoni* cercariae) and, 60 days post-infection, were ovalbumin (OA) immunised. The immunisation was also carried out in noninfected control offspring (uninfected control) (B). CFA: complete Freund’s adjuvant.

*Animals and S. mansoni infection -* Swiss Webster four-week-old female mice were infected subcutaneously (s.c.) with 20 *S. mansoni*cercariae, São Lourenço da Mata (SLM) strain. On the 45th day the infection was confirmed by Kato-Katz method (Katz et al. 1972). On the 60th dpi, estruses were synchronised by administration of 5 i.u. (100 mL) of equine chorionic gonadotrophin hormone plus, after 48 h, injection of an additional 5 i.u. (100 mL) of human chorionic gonadotrophin. The females were caged with male mice at a 1:1 ratio and successful mating was checked by presence of a vaginal plug. The same procedure was performed in noninfected females (Fig. 1A). Six-week-old offspring males were taken for the experimental and control groups. The mice were housed in the animal care facility at the Aggeu Magalhães Research Centre, Oswaldo Cruz Foundation (Fiocruz), municipality of Recife, state of Pernambuco, Brazil.

*Infection/immunisation protocol and study groups* - Immediately after birth, the newborns from *S. mansoni-*infected or noninfected mothers were housed in cages with interchanged mothers. After adoptive breastfeeding, offspring BIM were suckled by noninfected mothers and offspring SIM were suckled by infected mothers. Another group of animals was born and suckled by schistosomotic mothers (BSIM). Animals born from noninfected females were also suckled by their own mothers (control).

Six-week-old male offspring were infected with 80 *S. mansoni*cercariae, SLM strain (confirmed by Kato-Katz method) and, 60 dpi, immunised s.c. with 100 mg of OA (grade V; Sigma-Aldrich, USA) and emulsified in complete Freund’s adjuvant (CFA) (Sigma-Aldrich) at the base of the tail (0.1 mL/animal). The immunisation was also carried out in noninfected control offspring. Mice were divided into five groups (n = 10): (i) mice BIM; (ii) mice SIM; (iii) mice BSIM, (iv) mice control subsequently infected with *S. mansoni* and immunised with OA + CFA, (v) mice uninfected control and immunised with OA + CFA (Fig. 1B).

*Histomorphometric study of liver tissue* - On the 60th dpi, animals from BIM, SIM, BSIM, and control groups were immunised s.c and, nine days after immunisation, the parasite burden was determined and the livers were harvested after anaesthesia and euthanasia and fixed in 10% buffered formalin. Three fragments of liver tissue in transverse sections from three distinct lobes were collected from each animal. Horizontal histological sections (4 μm) were cut using a microtome Yamato (Japan) and the slides were stained with haematoxylin-eosin and Masson trichrome (selective for collagen) for morphometric study. The study was performed using ImageJ Software (National Institutes of Health, USA) for measuring the average diameter (micrometer - μm) of granulomas, with subsequent calculation of the area (μm2) and intensity of blue stain (specific for collagen) in histograms. Analyses were performed on images randomly obtained in 10-20 fields/animal (100X). The histomorphometric study was performed in five animals/group.

*Hypersensitivity reactions (HR)* - Immediate and cell-mediated HR in the different groups were elicited eight days after OA immunisation. Briefly, 30 μL of 2% aggregated OA was injected into one hind footpad and the same volume of saline in the other. Footpad swelling was periodically measured from 0.5-24 h using a pocket thickness gauge (Mitutoyo Mfg Co Ltd, Japan) and expressed as the increase in thickness relative to the saline-injected paw. The results are expressed as the median ± standard error (SE) for each group (n = 10). Mice nonimmunised with OA were equally challenged to test the controls for nonspecific swelling (data not shown).

*Detection of soluble egg antigen (SEA), soluble worm antigen preparation (SWAP), and OA-specific antibodies by ELISA* - *S. mansoni*SEA and SWAP were prepared as described by Boros and Warren (1970) and Pearce et al. (1988), respectively, and were used for parasite-specific antibodies production analysis. For heterologous antibody analysis was used OA. On the ninth day after immunisation, blood samples were taken by cardiac puncture from each group under intramuscular anaesthesia with xilazine HCl/ketamine HCl. Plasma samples were tested individually for IgG1 and IgG2a antibodies using SEA (1.25 μg/mL), SWAP (5 μg/mL), or OA (20 μg/mL)-coated 96-well plates (Nunc MaxiSorp, Denmark), and biotinylated goat antimouse IgG1 or IgG2a (Southern Biotechnology Associates Inc, USA). The reactions were developed with a streptavidin-peroxidase conjugate (Sigma-Aldrich) and an O-phenylenediamine (Sigma, USA) solution in 0.1 M citrate buffer plus H_2_O_2_. The plates were read (450 nm) in an automated ELISA reader. Titration curves were carried out for all the samples. The results are expressed as the median of the sample optical density from each group (n = 10) in an appropriated dilution (within the linear part of the titration curve) for each isotype ± SE (SEA 1:256 for IgG1 or 1:16 for IgG2a, SWAP 1:64 for IgG1 or 1:8 for IgG2a, OA 1:2.048 for IgG1 or 1:16 for IgG2a).

*Cell culture* - Nine days after immunisation, the spleen of each animal was harvested after euthanasia by cervical dislocation. Cell suspensions were prepared in RPMI-1640 (Sigma-Aldrich) supplemented with HEPES (10 mM), 2-mercaptoethanol (0.05 mM), 216 mg of L-glutamine/L, gentamicin (50 mg/L) and 5% of foetal bovine serum (FBS) (Sigma-Aldrich). The spleen cells from each group (n = 10) were cultivated at a final concentration of 10^7^ (24 h) or 6 × 10^6^ (72 h) cells/mL in 24-well tissue culture plates (Costar Culture Plates, USA) and subsequently stimulated with OA (500 μg/mL) or concanavalin-A (Con-A) (5 μg/mL) at 37ºC in 5% CO_2_. Supernatants were harvested after 24 h or 72 h and assayed for cytokine content: IL-4 (24 h), IFN-γ, and IL-10 (72 h). Cells cultured for 72 h were collected and labelled for CD4^+^ and FoxP3^+^ T-cells detection.

*Cytokine and CD4* ^*+*^ *FoxP3* ^*+*^ *T-cells measurements* - The cytokines were measured using specific two-site sandwich ELISA using the following monoclonal antibodies: for IFN-γ, XMG 1.2 and biotinylated AN18, for IL-4, 11B11 and biotinylated BVD6.24G2, and for IL-10, C252-2A5 and biotinylated SXC-1 (BD Biosciences Pharmingen, USA). Binding of biotinylated antibodies was detected using a streptavidin-peroxidase conjugate (Sigma-Aldrich) and a 2-2′-azinobis (3-ethylbenzene-thiazoline-6-sulphonic acid) (Sigma) solution in 0.1 M citrate buffer plus H_2_O_2_. The plates were read (405 nm) in an automated ELISA reader. The samples were quantified by comparison with the standard curves of purified recombinant cytokines (rIFN-γ, rIL-4, or rIL-10), with resulting detection limits of 2.5 ng/mL for IFN-γ and 0.3125 ng/mL for IL-4 or IL-10.

Spleen cells were subjected to double-labelling with fluorochrome-labelled antibody solutions at a concentration of 0.5 mg/10^6^ cells: PE antimouse FoxP3 plus PE-Cy5 antimouse CD4 (BD Biosciences Pharmingen). After staining, the preparations were washed with phosphate-buffered saline (PBS) containing azide (0.1%) and FBS (3%). After centrifugation, the cell pellet was resuspended in PBS with paraformaldehyde (0.5%) and maintained at 4ºC until the moment of data acquisition. Data acquisition was performed using a flow cytometry FACSCalibur (BD-Pharmingen, USA) by collecting a minimum of 10,000 events per sample. The frequency of positive cells was analysed using the program Cell Quest Pro and the limits for the quadrant markers were always set based on negative populations and isotype controls. A descriptive analysis of the frequency of cells in the upper right quadrant (double-positive cells) was performed. The results are expressed as the mean of the frequency of cells double-labelled from each group ± standard deviation.

*Statistical analysis* - For HR analysis, the Wilcoxon test (treatment × time) was used to evaluate the differences among groups, whereas for antibody production and histomorphometric analysis of liver sections, the Kruskal-Wallis test. The multiple comparisons were performed by Mann-Whitney *U*test. For cytokine analysis and flow cytometry, an one-way analysis of variance followed by Tukey’s method were used. For statistical analysis, we used GraphPad Prism v.5.0 (GraphPad Software, USA) and all findings were considered significant at p < 0.05. All procedures were repeated three times to evaluate the reproducibility of the results and it was showed one representative of three independent studies.

*Ethics* - The animal protocol was approved by the Ethical Commission on Animal Use of the Fiocruz (L-0063/08) and is in accordance with the Ethical Principles in Animal Research adopted by the Brazilian College of Animal Experimentation.

## RESULTS

**Fig. 2:**
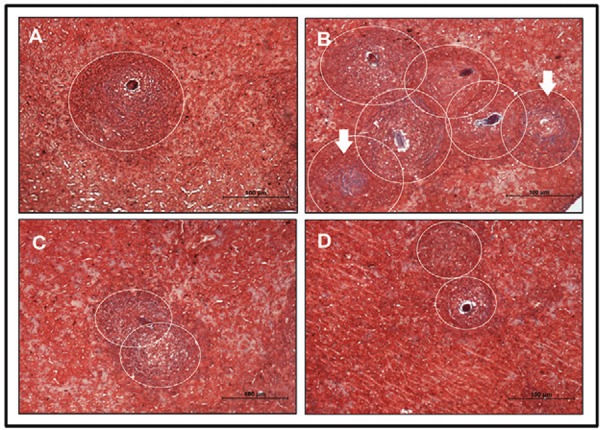
: histomorphometric study of liver tissue. Analysis of hepatic granulomas in Swiss Webster mice born and suckled from uninfected mothers (control) (A), born from infected mothers (B), suckled by infected mothers (C), and born and suckled by schistosomotic mothers (D) and 69th day post-infection with 80 *S. mansoni*cercariae. The slides were stained with Masson trichrome (selective for collagen) and the analyses were performed on images randomly obtained in 10-20 fields/animal (100X). The histomorphometric study was performed in five animals/group. Circles defining the granuloma size (A, B, C and D) and arrows indicate areas with increased collagen deposition (B).

*BIM mice show more intense granulomatous reactions, whereas previous intake of breast milk from schistosomotic mothers strongly reduced hepatic inflammation* - To verify the intensity of the granulomatous reaction, a hepatic histomorphometric analysis was performed on the 69th dpi. We noted significantly more granulomas, including a greater quantity of collagen in the BIM group compared to the control group (Fig. 2B,Table). The livers of mice that received breast milk from infected mothers (SIM and BSIM) showed a similar number of granulomas and collagen quantity compared with the control group; however, the granuloma sizes were smaller (Fig. 2C, D, Table). Additionally, we observed no significant difference in the number of eggs in faeces or worm recovery of BIM or SIM descendants compared with the control group (Table). By contrast, in BSIM animals there was a 72% reduction in the quantity of eggs per gram of faeces and 54% reduction in the worm numbers. It was also observed in mice that received maternal milk a lower number (BSIM groups) and less size of granulomas (SIM and BSIM groups) in the intestinal histomorphometric analysis (data not shown).

**TABLE:** Number and size of granulomas and hepatic fibrosis developed in mice infected with 80 *Schistosoma mansoni* cercariae born from infected mothers (BIM), suckled by infected mothers (SIM), and born and suckled by schistosomotic mothers (BSIM)

Groups^*a*^	Kato-Katz (epg)^*b*^	Worm recovery^*c*^	Number of hepatic granulomas^*d*^		Granuloma size^*e*^ (MT)	Collagen^*f*^ (MT)

			(H&E)	(MT)		
Control	509.1 ± 359.1	15.00 ± 1.83	2.89 ± 1.22	3.13 ± 1.95	23237 ± 7934	78.67 ± 6.64
BIM	408.0 ± 159.2	10.14 ± 2.27	4.63 ± 1.38^*g*^	5.73 ± 2.25^*g*^	21451 ± 9454	87.46 ± 10.46^*g*^
SIM	332.6 ± 202.7	12.67 ± 3.20	3.11 ± 1.07	3.84 ± 2.21	18407 ± 6674^*g*^	79.33 ± 7.98
BSIM	142.3 ± 103.7^*g*^	6.88 ± 3.44^*g*^	3.74 ± 1.45	4.16 ± 2.10	20467 ± 8577^*g*^	83.91 ± 9.01

*a:* Swiss Webster mice infected with 80 *S. mansoni* cercariae, BIM, SIM, or BSIM from *S. mansoni* infected mothers immunised subcutaneously with ovalbumin (100 μg/animal) in complete Freund`s adjuvant 60 days post-infection had their liver subjected to morphometric study nine days after immunisation. Born and suckled mice from uninfected mothers (control) were also analysed under the same conditions [haematoxylin-eosin (H&E) and Masson’s trichrome (MT) stains];*b*: median ± standard error (SE) of number of the eggs per gram (epg) faeces. Analysis was conducted 49 days post-infection; *c*: median ± SE of parasite burden. Analysis was conducted 69 days post-infection; *d*: median ± SE of number of hepatic granulomas per field;*e*: median ± SE of size (sectional area) of hepatic granulomas in µm^2^. Analysis of 20 granulomas per animal (n = 5) totalling 100 granulomas/group; *f*: median ± SE of proportion of collagen obtained from the ImageJ^®^ software; *g*: p < 0.05 compared with the control group.

**Fig. 3:**
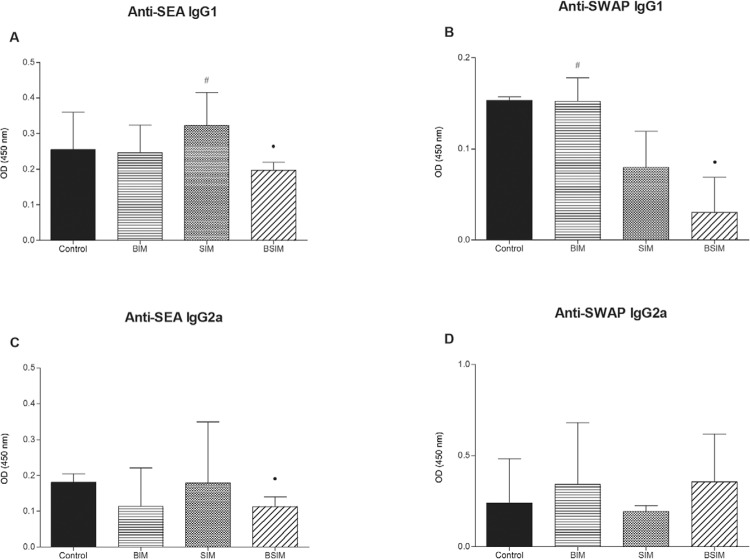
: soluble egg antigen (SEA) and soluble worm antigen preparation (SWAP)-specific IgG1 (A and B) and IgG2a (C and D) antibodies. Swiss Webster mice born from infected mothers (BIM), suckled by infected mothers (SIM), born and suckled by schistosomotic mothers (BSIM), and born and suckled from uninfected mothers (control) were postnatal infected with 80 *S. mansoni* cercariae. Isotype levels in the plasma were measured by ELISA in dilutions SEA 1:256 (IgG1) and 1:16 (IgG2a), and SWAP 1:64 (IgG1) and 1:8 (IgG2a) 60 days after infection. The results represent the median of absorbance (OD) ± standard error for 10 animals/group. The results are showing one representative of three independent experiments. •: p < 0.05 compared with the control group; #: p < 0.05 compared with the BSIM group.

*Descendants BSIM had lower levels of anti-SEA and anti-SWAP antibodies* - When analysing anti-SEA (Fig. 3A, C) and anti-SWAP (Fig. 3B, D) IgG1 and IgG2a production in the descendants of schistosomotic or uninfected mothers after infection as adults, we observed decreased production of anti-SEA IgG1 and IgG2a and anti-SWAP IgG1 in the BSIM group compared to the control group. Furthermore, we observed greater production of anti-SEA IgG1 in the SIM group and anti-SWAP IgG1 in the BIM group compared to the BSIM group.

*Anti-OA immediate HR are suppressed during postnatal infection, mainly in descendants SIM* - The BIM, SIM, BSIM, and control groups of mice were subjected to postnatal *S. mansoni* infection and, 60 days later, were immunised with OA in adjuvant. To evaluate in vivo anti-OA HR, all groups were challenged with OA aggregates in the footpad and swelling was measured. The same analysis was performed in newborns born/suckled by noninfected mothers that were not infected (uninfected control).

**Fig. 4:**
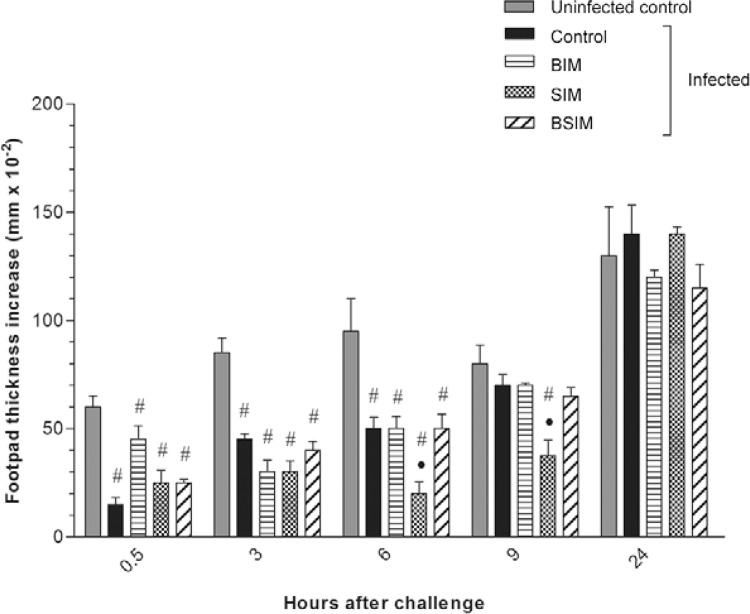
: hypersensitivity reactions to ovalbumin (OA). Swiss Webster mice born from infected mothers (BIM), suckled by infected mothers (SIM), and born and suckled by schistosomotic mothers (BSIM) were immunised with OA (100 μg/animal) in complete Freund’s adjuvant 60 days after postnatal infection (80 *Schistosoma mansoni* cercariae). All the mice were challenged with OA aggregated in the footpad eight days after immunisation. Postnatal infected or uninfected mice born and suckled by uninfected mothers (control) were also immunised with OA and equally challenged. The results represent the median of the net increase in footpad thickness of 10 mice/group ± standard error. The results are showing one representative of three independent experiments. •: p < 0.05 compared with the control group; #: p < 0.05 compared with the uninfected control group.

As shown in Fig. 4, all infected groups had immediate HR (at 0.5-6 h) that were significantly lower than that in the uninfected group (uninfected control). At 6 h, the SIM group showed significantly less HR than the group of infected descendants from nonschistosomotic mothers (control). This suppression was also observed at 9 h in the SIM group.

Delayed anti-OA HR (24 h) was observed in uninfected control group and was similar to groups of infected descendants from nonschistosomotic and schistosomotic mothers.

**Fig. 5:**
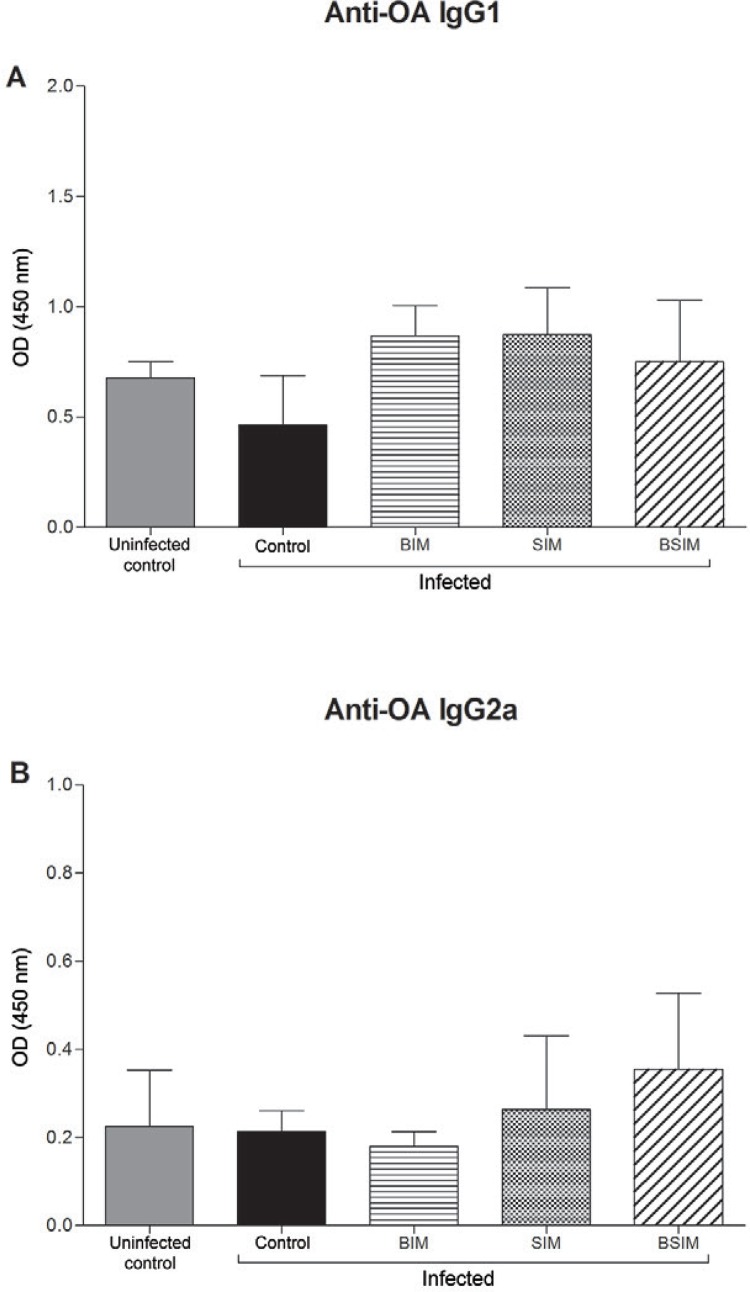
: ovalbumin (OA)-specific IgG1 (A) and IgG2a (B) antibodies. Swiss Webster mice born from infected mothers (BIM), suckled by infected mothers (SIM), and born and suckled by schistosomotic mothers (BSIM) were immunised with OA (100 μg/animal) in complete Freund’s adjuvant 60 days after postnatal infection (80 *Schistosoma mansoni*cercariae). Postnatal infected or uninfected mice born and suckled by uninfected mothers (control) were also immunised with OA. Isotype levels in the plasma were measured by ELISA in dilutions 1:2.048 (IgG1) and 1:16 (IgG2a) nine days after immunisation. The results represent the median of absorbance (OD) ± standard error for 10 animals/group. The results are showing one representative of three independent experiments.

*S. mansoni infection did not change the production of anti-OA IgG1 and IgG2a in the descendants of schistosomotic mothers as adults* - Nine days after immunisation, all groups were bled, and the serum antibody levels were measured by ELISA. Anti-OA IgG1 (Fig. 5A) and IgG2a (Fig. 5B) levels were similar between all of the groups infected as adults and the uninfected control group.

**Fig. 6:**
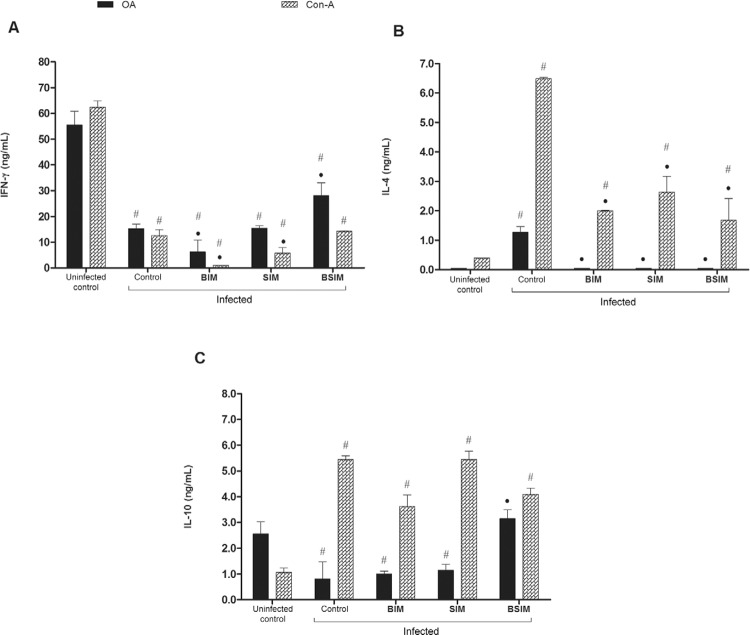
: interferon (IFN)-γ (A), interleukin (IL)-4 (B), and IL-10 (C) secreted by spleen cells. Swiss Webster mice born from infected mothers (BIM), suckled by infected mothers (SIM), and born and suckled by schistosomotic mothers (BSIM) were immunised with ovalbumin (OA) (100 μg/animal) in complete Freund`s adjuvant 60 days after postnatal infection (80 *Schistosoma mansoni* cercariae). Postnatal infected or uninfected mice born and suckled by uninfected mothers (control) were also immunised with OA. On 9th day, 107 cells (IL-4) or 6 x 106 cells (IFN-g and IL-10) were stimulated with OA (500 μg/ml) or concanavalin-A (Con-A) (5 μg/mL) for 24 h (IL-4) or 72 h (IFN-g and IL-10). Cytokines were quantified in supernatants harvested by sandwich ELISA. The results represent the mean ± standard deviation for 10 animals/group. Nonstimulated cells produced < 2.5 ng/mL of IFN-g, < 0.44 ng/mL of IL-4, and < 0.625 ng/mL of IL-10. The results are showing one representative of three independent experiments. •: p < 0.05 compared with the control group: #: p < 0.05 compared with the uninfected control group.

*IFN-*g*, IL-4, and IL-10 production in adult descendants of schistosomotic mothers after postnatal infection* - Splenocytes from animals of the groups studied were cultured in the presence of OA or Con-A and the supernatants were collected to measure the secreted cytokines. In these conditions, IFN-g production was significantly lower in all infected groups compared with the uninfected control group (Fig. 6A). However, in animals born from schistosomotic mothers, there was also a lower quantity of this cytokine compared with the control group. This finding was observed in the group that received only breast milk from schistosomotic mothers and was cultured with a mitogenic stimulus. In the BSIM group, the production of IFN-g in response to OA was significantly higher than in the control animals.

IL-4 production, in an in vitro response to OA, was only detected in the supernatant of splenocytes from control animals (Fig. 6B). In response to mitogen, all groups of infected animals produced significantly more IL-4 than the uninfected control group. Still, in the groups of descendants from schistosomotic mothers, IL-4 production was significantly lower than in the group of infected descendants from noninfected mothers (control).

For IL-10 production after Con-A stimulation, the infected mice produced significantly higher levels of this cytokine than the uninfected control mice (Fig. 6C). In response to OA, the production of IL-10 was significantly lower in infected animals (control) and in animals that were born (BIM) or breastfed (SIM) by schistosomotic mothers compared with the uninfected control mice. By contrast, there was a higher production of this cytokine in the BSIM group compared with the control group.

**Fig. 7:**
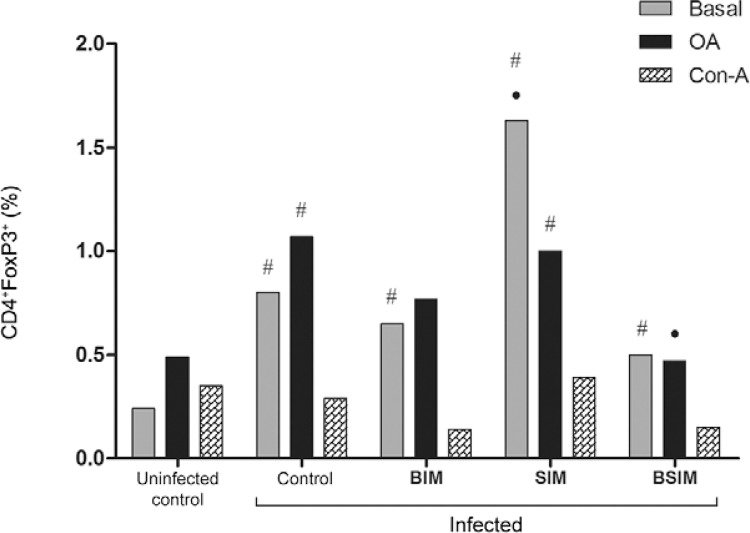
: splenic cells expressing CD4+FoxP3+. Swiss Webster mice born from infected mothers (BIM), suckled by infected mothers (SIM), and born and suckled by schistosomotic mothers (BSIM) were immunised with ovalbumin (OA) (100 μg/animal) in complete Freund’s adjuvant 60 days after postnatal infection (80 *Schistosoma mansoni* cercariae). Postnatal infected or uninfected mice born and suckled by uninfected mothers (control) were also immunised with OA. Nine days after immunisation, their spleen cells were unstimulated or stimulated with OA (500 μg/mL) or concanavalin-A (Con-A) (5 μg/mL) for 72 h, labelled and analysed by flow cytometry. The results represent the mean of the frequency of spleen cells double-labelled ± standard deviation for 10 animals/group. The results are showing one representative of three independent experiments. •: p < 0.05 compared with the control group; #: p < 0.05 compared with the uninfected control group.

*Postnatal infection lead to increased CD4* ^*+*^ *FoxP3* ^*+*^ *T-cells frequencies in adult descendants, mostly in SIM mice* - In unstimulated cultures, the infected groups showed a higher frequency of CD4^+^FoxP3^+^ T-cells compared with the uninfected control group (Fig. 7). When only the infected groups were compared, the SIM mice showed the highest CD4^+^FoxP3^+^T-cells frequency (BIM = 0.65%, SIM = 1.63%, BSIM = 0.50%, control = 0.80%, and uninfected control = 0.25%). In Con-A-stimulated cultures, the frequencies of CD4^+^FoxP3^+^ T-cells were similar (BIM = 0.14%, SIM = 0.39%, BSIM = 0.15%, control = 0.29%, and uninfected control = 0.35%). In OA-stimulated cultures, the infected SIM and control groups showed an increased frequency of CD4^+^FoxP3^+^ T-cells compared with the uninfected control group (BIM = 0.77%, SIM = 1%, BSIM = 0.47%, control = 1.07%, and uninfected control = 0.49%). Splenocytes from the BSIM group that were stimulated with OA showed a significantly lower frequency of CD4^+^FoxP3^+^ T-cells compared with the control group.

## DISCUSSION

In this study we evaluated the effect of pregnancy, separately from the breast milk, on the intensity of the hepatic granulomatous reaction, as well as on immunity to heterologous antigen during the postnatal infection of descendants from schistosomotic mothers. For comparison, group of mice that were born and suckled by noninfected mothers were postnatally infected. As expected, in infected descendants from noninfected mothers we observed the hepatic granulomas induced by *S. mansoni* eggs, such as skewing of the immune response to a heterologous antigen towards a Th2/IL-10 profile (IL-4 and IL-10, suppression of immediate HR, IFN-g production, and a optimal frequency of CD4^+^FoxP3^+^T-cells) (Stavitsky 2004, Gryseels et al. 2006, Barsoum et al. 2013, Lundy & Lukacs 2013). However, this scenario was altered by previous contact with schistosomotic mothers, either *in utero* or through breast milk.

BIM group presented greater quantity of hepatic granulomas and remarkable fibrosis intensity, which can be due to uterine conditions in infected mothers. It is widely accepted that alternatively activated macrophages (M2) are induced in the uterine mucosa to favour foetal tolerance (Gustafsson et al. 2006, Svensson et al. 2011) and are committed to tissue remodelling (Gordon & Martinez 2010). In infected mothers, these conditions could be amplified by *S. mansoni* antigens which are strong inducers of M2 (Wilson et al. 2007, Joshi et al. 2008) and imprint long-term predisposition for collagen production in adult life. Currently, this hypothesis is being tested in our experimental model. Postnatal infection of animals pre-exposed to parasite antigens*in utero* strongly impairs both anti-OA Th1/IFN-g and Th2/IL-4 responses. Recently, we reported reduced expression of the co-stimulatory molecule CD86 in response to OA by CD11c^+^ cells of adult animals born from infected mothers (Santos et al. 2014) that may have reduced capacity to prime anti-OA Th1 and Th2 responses.

In descendants that had ingested the breast milk of infected mothers, the granulomatous reaction was markedly reduced, analogous to the changes in anti-OA Th1 and Th2 immunity. In the cell culture of these offspring, there was a remarkable background level for CD4^+^FoxP3^+^ T-cells. Although, we would have to label more molecules to confirm the Treg cells phenotype (von Boehmer 2005, Taylor et al. 2006, Collison et al. 2009), these basal conditions could downregulate the immune responses to both homologous (McKee & Pearce 2004,Taylor et al. 2006, Wilson et al. 2007) and heterologous antigens (Smits et al. 2007, Cardoso et al. 2012). Whether parasite antigens in the breast milk in contact with the suppressive intestinal mucosa microenvironment (Weiner et al. 2011) may collaborate for enhanced CD4^+^FoxP3^+^ T-cells generation after antigenic re-exposure as adults deserve further investigations. Intriguingly, in SIM mice, we observed higher CD40^+^CD80^+^ B-cells frequency and greater IL-2 production after OA stimulation (Santos et al. 2010, 20, 2014), which are conditions to favour increased frequency of CD4^+^FoxP3^+^ T-cells (Zheng et al. 2010). Besides of this, the contact with anti-SEA antibodies at a young age can generate idiotypes and antiidiotypes that negatively modulate granulomatous reactions (Caldas et al. 2008), and only passive transfer by breastfeeding, but not pregnancy, maintains the levels of anti-SEA IgG1 in the early in life (Nóbrega et al. 2012). Therefore, the modulation of granuloma by this mechanism must not be too ruled out in the SIM group.

Attallah et al. (2006) and Othman et al. (2010) previously reported a reduced granulomatous reaction in animals born and suckled by infected mothers with a high parasite load. Here, the results in BSIM group corroborated these data and showed that continuous contact with parasite antigens during breastfeeding in infected mothers reverted the strong hepatic damage that was obtained in prenatal phase. In our study, this phenomenon was achieved using a low maternal parasite load, which is similar to the conditions of endemic populations in the Northeast Region of Brazil (Tanabe et al. 1997, daFrota et al. 2011). Othman et al. (2010) showed increased levels of the cytokines IL-12 and TGF-β, which can counteract immune regulation. In our studies, we observed, in the cell culture, increased IL-10 (upon mitogenic stimulus) and CD4^+^FoxP3^+^ T-cells. Taken together, these findings corroborate that controlled Th1 and Th2 responses is required to minimise the severity of the hepatic pathology (Wilson et al. 2007). Besides of this, in BSIM group there was reduction in the eggs quantity and worm numbers. This finding reflects concomitant immunity, in which adult worms and egg antigens stimulate a protective immune response against new infections (Salim & Al-Humiany 2013) by reaching the intestinal epithelium (Schramm & Haas 2010). This situation mimics the antigens present in the breast milk that continuously act during lactation.

For anti-OA immune responses, there was a partial recovery of anti-OA immunity in animals from the BSIM group, which was observed by increased IFN-g and IL-10 levels. Thus, these cytokines may be more involved with the reduced IL-4 levels in BSIM animals, since anti-OA CD4^+^FoxP3^+^ T-cells were found at a lower frequency in this group compared to the infected control group.

In regard to the antibodies against parasite antigens, our results were in agreement to study of the Attallah et al. (2006), in which were detected low levels of antibodies in the BSIM group in comparison to control. However, in postnatal infection of offspring that breastfed or pregnancy in separate way, anti-SEA and anti-SWAP IgG1 levels were similar to control group, respectively.

The immunomodulatory actions of infection on heterologous humoral immune responses are contradictory (Kullberg et al. 1992,Curry et al. 1995, Montesano et al. 1999, Smits et al. 2007, Cardoso et al. 2010). In this study, there was no significant difference in the levels of anti-OA IgG1 and IgG2a in the groups. However, some considerations about the BIM and SIM groups must be highlighted. Previously, we have shown an increase in anti-OA antibody levels in the SIM group (Santos et al. 2010), and those with postnatal infection showed similar levels to the uninfected control group. Therefore, this reduction of the anti-OA immune response supports the immunosuppressive profile that results from postnatal infection in the SIM group. In the BIM group, there was reduced anti-OA antibody production (Santos et al. 2010), which was recovered after infection of the mice as adults. Thus, the levels of anti-OA antibodies were similar between groups. These data could reflect the control of immunologic diseases that are mediated by antibodies, such as allergies and autoimmune diseases.

In conclusion, congenital exposure to *S. mansoni* antigens favoured exacerbated immunopathology in postnatal infections. However, when it was immediately followed by breastfeeding, the chronic hepatic disease was controlled and there was partial restoration of the heterologous anti-OA immunity (IFN-g production). Based on these results, we suggest that nonadoptive breastfeeding, i.e., from the biological mother, is more effective for immunomodulation of the granulomatous reaction in individuals from endemic areas who are at risk of postnatal infection. Nonadoptive breastfeeding can also guarantees better protection for nonrelated antigens, infections, and responses to vaccines, in these individuals.
